# Single-port laparoscopy: Considerations in children

**DOI:** 10.4103/0972-9941.72395

**Published:** 2011

**Authors:** Todd A Ponsky, David M Krpata

**Affiliations:** Department of Pediatric Surgery, Rainbow Babies and Children’s Hospital, 11100 Euclid Ave, Cleveland, OH 44106, USA

**Keywords:** Minimally invasive surgery, paediatrics, single-access surgery, single-port surgery

## Abstract

As the quest to minimize scars from surgery continues, innovative methods of surgery, including single-port surgery, have come to the forefront. Here, we review considerations for surgery in children with particular attention to appendectomy and cholecystectomy. We discuss the future technologies that will aid in single-port surgery and how they apply to the paediatric population.

## INTRODUCTION

In the continuous quest to minimize the scars from surgery, surgeons have recently explored new innovative solutions. Specifically, single-port laparoscopic surgery has gained immense popularity throughout the world within the past 5 years. The theoretical appeal of this technique is that scars can be hidden with the natural scar of the patient, the umbilicus. Although technology has not yet caught up with the technique, surgeons and patients are becoming more and more interested in this technique. Although most of these procedures are being performed in adult patients, paediatric surgeons are also implementing this into their practice. We first reported our experience with this technique in children.[1] Since then, numerous reports of single-port surgery in children have been published. While most of the tools and techniques of single-port surgery are similar between adults and children, here, we describe some of the differences and special considerations.

### Paediatric single-port procedures

Similar to adults, the most commonly performed single-port operations in children are probably the cholecystectomy and the appendectomy. Other procedures include, but are not limited to, intussusception, splenectomy, inguinal hernia repair, thoracic decortication for empyema, lung biopsy, nissen fundoplication, gastrostomy tube placement and pyloromyotomy.

## CHOLECYSTECTOMY

### Indications

At our institution, similar to the adult population, the most common indication for single-port cholecystectomy is symptomatic cholelithiasis followed by biliary dyskinesia. However, many of our patients who require cholecystectomy are sickle cell patients. These are often small children who have developed bilirubinate stones. Acute cholecystitis is rare at our institution.

### Operative Considerations

In our practice, the size of the child often determines the technique used. For instance, in smaller children, we prefer the multiple trocar technique. The use of multiple trocars usually allows for a smaller incision as opposed to the multiple-port trocars. In larger children, we prefer the single, muliport trocars. Patients are placed in the split-legged (French) position. The surgeon stands between the legs and the assistant stands to the patients left.

The operation begins by injecting 0.25% bupivicaine into the inferior portion of the umbilicus. A 15-mm incision is created in an infraumbilical fashion. A vertical incision can also be performed through the umbilicus, but the authors believe that the infraumbilical incision is cosmetically superior. If multiple trocars are being used, a veress needle is inserted and the abdomen is insufflated to 15 mmHg. An optical Surgiquest Anchorport trocar is then inserted through the superior, middle portion of the umbilical incision. A camera is inserted to assure that the abdominal contents have safely fallen away from the abdominal wall prior to inserting more trocars. Next, two subsequent trocars are placed on either side of the initial trocar. The camera is inserted through the 5 o’clock port. We typically use a 50-cm high-definition laparoscope with a right angle-like cord adapter (Storz, Tuttlingen, Germany) or the 45 cm scope from Stryker Endoscopy (San Jose, CA, USA). After the camera is inserted, a 2 mm Minilap Gator grasper (Stryker Endoscopy) is inserted through the inferior portion of the incision. This grasper is used to retract the dome of the gallbladder in a cephalad position and is clamped down to the drapes such that it is out of the way for the entire case. Next, a reticulating grasper is used to retract the infundibulum to the right and is slightly cephalad. This Endograsper (Covidien, Mansfield, MA, USA) is usually placed in the 2 o’clock port. Once reticulated, the handle of the grasper rotates to the surgeon’s right side, away from the other instruments. The surgeon stands between the legs of the patient while the assistant stands to the right of the surgeon on the other side of the left leg. The assistant holds the scope and the grasper while the surgeon uses two hands on the dissector. A dissector is then placed through the 10 o’clock port. We typically begin the operation with a straight Maryland dissector or a hook cautery. If, during the operation, there is poor visibility because the camera is staring down the shaft of the instrument or because there is a strain on the instrument to reach the area of dissection, we switch to a reticulating instrument. When a reticulating instrument is used, we prefer the RealHand hook cautery (Novare Surgical). If possible, the lateral attachments of the gallbladder to the liver are divided first to lengthen the cystic duct and cystic artery before initiating dissection at the cystic duct. Once the cystic duct is clearly dissected free, it is doubly ligated with clips. The duct is then divided and our attention is turned to the cystic artery. We doubly ligate the artery with clips and divide it with laparoscopic shears. Once the cystic duct and artery are divided, the gallbladder is then removed from the gallbladder fossa with cautery. Next, the three 5-mm trocar incisions are connected with cautery and the gallbladder is removed through this incision without the use of an endobag. The incision is typically 1.5–2 cm and is then closed with a vicryl stitch in the skin, closed with an absorbable subcuticular stitch.

For larger patients, we use one, multiport trocar such as the SILS™ port from Covidien. This trocar necessitates a slightly larger incision than using multiple trocars, but gives more freedom of movement and minimal air leakage. This technique begins by entering the abdomen using the Hassan technique and inserting the foam trocar into the incision. The trocar is rotated so that there is a port at the 10, 5 and 2 o’ clock positions and the rest of the operation is performed as described above. The Minilap gator is inserted within the umbilical incision, alongside the trocar, at the 7 o’ clock position.

## APPENDECTOMY

### Indications

At our institution, appendectomy is the most common single-port operation performed. Most of our patients are teenagers and the majority have nonperforated appendicitis. A recent trend in paediatric surgery is the initial nonoperative management of perforated appendicitis followed by interval appendectomy. For this reason, single-port appendectomy for ruptured appendicitis is rare at our institution.

## OPERATIVE CONSIDERATIONS

### Extra-corporal, single-port, appendectomy

Although single-port surgery has gained recent widespread popularity, single-port appendectomies in children have been described for many years.[2] This technique was originally described using an operative laparoscope to exteriorize the appendix through the umbilicus and perform an extra-corporeal appendectomy. With the advent of newer operative laparoscopes and the new interest in single-port laparoscopy, this technique has become more popular.

Recently, similar alternatives have been developed to exteriorize the appendix without an operative laparoscope. Although not yet published, Kanard *et al*. recently described his technique at The International Pediatric Endosurgery Group in June, 2010, in Hawaii, in which he placed a 3-mm instrument alongside a 5-mm camera port and enlarged the fascial incision once the appendix had been grasped. At the same meeting, Guelfand *et al*. described a trocarless technique in which the camera, insufflation tubing and a 5-mm instrument are placed through an umbilical incision.

This technique may be unique to children because the thin abdominal wall and shorter distance from the cecum to the umbilicus allows for the appendix to be easily exteriorized. However, we have used this technique, although with more difficulty, even in larger adolescents. While some argue that this technique may lead to an increase in umbilical wound infections. This has not yet been shown.

### Intracorporal, single-port, appendectomy

Another alternative for performing single-port appendectomies is the intracorporeal technique. In this technique, three individual trocars are placed in the umbilicus or one multi-port trocar is placed and the mesoappendix and appendix are divided intracorporeally. Some have described only using a telescope and one instrument while the appendix is retracted using a transcutaneous stitch.[3] Suttie *et al*. compared the outcomes from the intra- and extracorporeal techniques and found a slight increase in wound infections in the extracorporeal group, but with faster operative times.[4] Unfortunately, this study was low powered.

### Other Procedures

Other single-port procedures that have been performed in children include intussusception reduction, Nissen fundoplication, ovarian detorsion, thoracoscopic decortication, lung biopsy, inguinal hernia, gastrostomy tube placement, splenectomy, gastric sleeve resection and gastric band placement. Most of these techniques are similar to the adult counterpart.

### Emerging Technology

Inherently, new technologies advance to improve the technologies’ shortcomings as we begin employing them. Single-site surgery is no different and the future offers advancements that will improve visualization, retraction, triangulation and, ultimately, patient safety. One of the most promising advancements is the use of flexible laparoscopy, which uses flexible instruments to improve triangulation. The Spider surgical system (TransEnterix, Durham, NC, USA), is a single-access device that has two flexible channels which deploy like an umbrella after insertion into the abdomen. This allows the surgeon to use two flexible laparoscopic instruments and improve triangulation.

Advancements in the field have also allowed for improved retraction using noninvasive techniques. Dr. Marcelo Martinez Ferro and his group in Argentina have recently presented their work using magnet surgical devices to aid in retraction during a single-site laparoscopic Nissen fundoplication. This technology uses intracorporeal graspers with magnets and an extracorporeal magnet that can be manipulated over the abdominal wall to maximize retraction.

As new technologies emerge, it is important to remember that using additional ports is not a failure of single-port surgery. Needlescopic surgical devices, instruments that are 2–3 cm in diameter, will play an important role in the future of minimally invasive surgery as these allow the addition of instruments without the amount of abdominal wall trauma that traditional laparoscopic instruments cause. Instruments such as the Minilap (Stryker Endoscopy), which we use in our single-port cases already, allow for percutaneous introduction of instruments to aid in dissection, retraction and visualization when difficulties are encountered during single-port surgery. Given the thinner abdominal wall of children, needlescopic instruments should play an even more prominent role than in the adult population.

## CONCLUSION

As the surgical field continues the quest to minimize patient scars and maximize patient safety, new technologies will continue to emerge. I would expect that single-site surgery will continue to gain popularity and may even one day become the standard of care in adults and paediatrics. Single-port surgery in children is a feasible option. It is critical to remember that we are in the early stages of single-port surgery and that patient selection plays a significant role in maximizing patient safety. The future of single-port surgery is bright and this is an exciting time for the field of surgery.

## References

[1] Ponsky TA, Diluciano J, Chwals W, Parry R, Boulanger S (2009). Early experience with single-port laparoscopic surgery in children. J Laparoendosc Adv Surg Tech A.

[2] Esposito C (1998). One-trocar appendectomy in pediatric surgery. Surg Endosc.

[3] Ateş O, Hakgü;der G, Olguner M, Akgür FM (2007). Single-port laparoscopic appendectomy conducted intracorporeally with the aid of a transabdominal sling suture. J Pediatr Surg.

[4] Suttie SA, Seth S, Driver CP, Mahomed AA (2004). Outcome after intra- and extra-corporeal laparoscopic appendectomy techniques. Surg Endosc.

